# Supplementary material for: Biodegradation Mechanism of Polystyrene by Mealworms (Tenebrio molitor) and Nutrients Influencing Their Growth
Source: Polymers (Basel). 2024 Jun 9;16(12):1632. doi: 10.3390/polym16121632 (PMC11207799; doi:10.3390/polym16121632)
Supplement: Supplementary file 1 [file polymers-16-01632-s001.zip › polymers-3029673-supplementary.pdf]

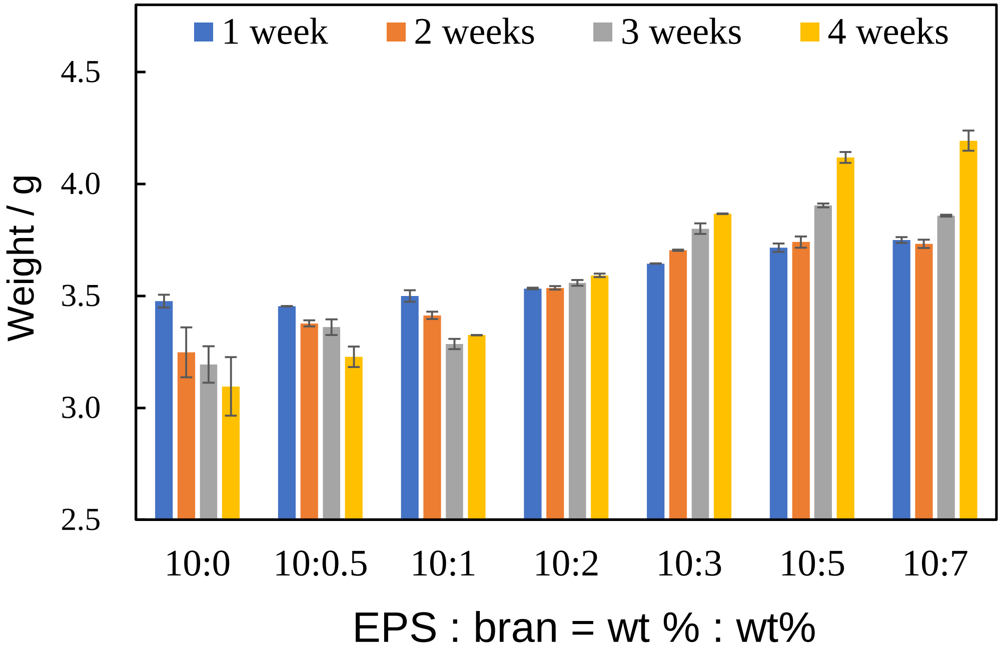

Figure S1 Dependence of mealworm weight (total 30 worms) on rearing period in EPS and bran mixed diets with different mixing ratios

P (%)	O (%)	C (%)	Mg(%)
2.26	28.36	68.38	1.00

% : mole percent

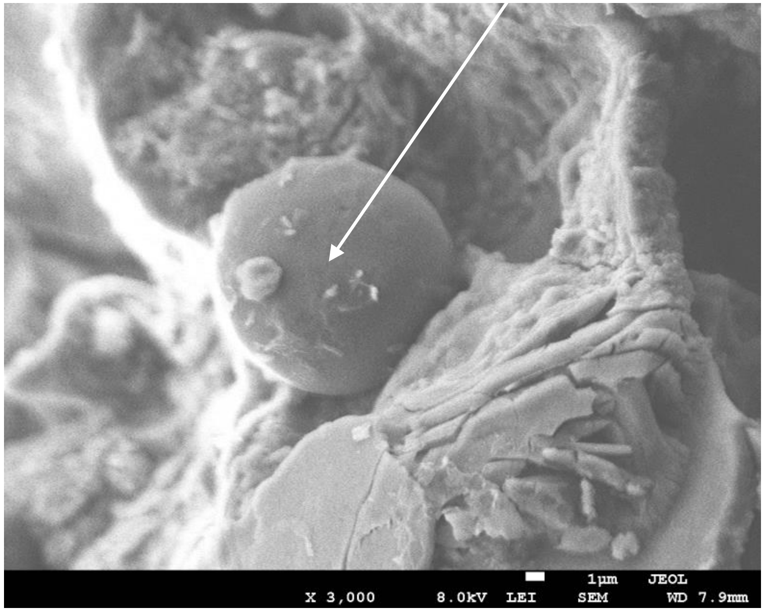

Figure S2 SEM/EDX analysis of frass passed from the body of a mealworm fed only bran as feed after 1 week of rearing.

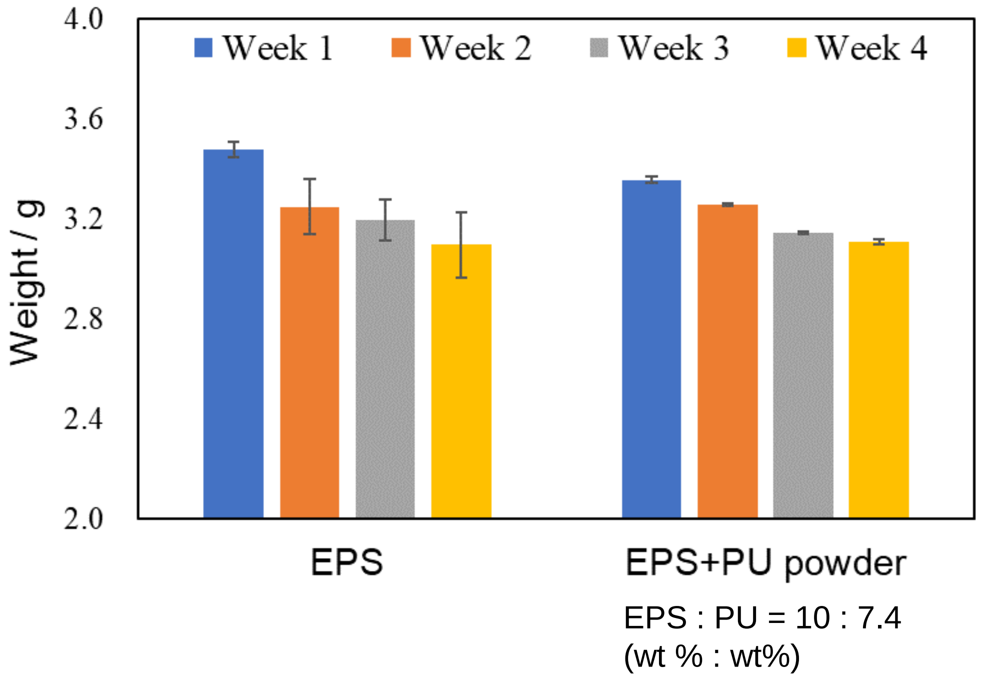

Figure S3 Dependence of mealworm weight (total 30 worms) on rearing period in EPS and EPS + PU powder mixed diets.
